# Physicochemical Profiling, Bioactive Properties, and Spectroscopic Fingerprinting of Cow’s Milk from the Pampas Valley (Tayacaja, Peru): A Chemometric Approach to Geographical Differentiation

**DOI:** 10.3390/molecules30224484

**Published:** 2025-11-20

**Authors:** Eudes Villanueva, Harold P. J. Ore-Quiroz, Gino P. Prieto-Rosales, Raquel N. Veliz-Sagarvinaga, Yaser M. Chavez-Solano, Elza Aguirre, Gustavo Puma-Isuiza, Beetthssy Z. Hurtado-Soria

**Affiliations:** 1 Departamento Académico de Ingeniería en Industrias Alimentarias, Universidad Nacional Autónoma de Tayacaja Daniel Hernández Morillo (UNAT), Jr. Bolognesi Nro. 418, Pampas 09156, Peru; haroldore@unat.edu.pe (H.P.J.O.-Q.); ginoprieto@unat.edu.pe (G.P.P.-R.); beetthssy.hurtado@unat.edu.pe (B.Z.H.-S.); 2 Escuela Profesional de Ingeniería en Industrias Alimentarias, Universidad Nacional Autónoma de Tayacaja Daniel Hernández Morillo (UNAT), Jr. Bolognesi Nro. 418, Pampas 09156, Peru; 75089154@unat.edu.pe (R.N.V.-S.); 71374282@unat.edu.pe (Y.M.C.-S.); 3 Instituto de Investigación Tecnológica Agroindustrial, Universidad Nacional del Santa (UNS), Av. Universitaria s/n, Nuevo Chimbote 02712, Peru; eaguirre@uns.edu.pe; 4 Facultad de Industrias Alimentarias, Universidad Nacional Agraria La Molina (UNALM), Av. La Molina s/n, Ap. 12056, Lima 150114, Peru; gpuma@lamolina.edu.pe

**Keywords:** Raman spectroscopy, infrared spectroscopy, chemometrics, fatty acids, amide I, molecular vibration

## Abstract

This study aimed to characterize the physicochemical and functional properties of bovine milk from four districts (Acraquia, Ahuaycha, Pampas, and Daniel Hernández) of the Pampas Valley, Tayacaja province, Huancavelica (Peru), and assess its geographical traceability using vibrational spectroscopy and chemometric tools. Milk samples were analyzed for composition (fat, protein, lactose, salts), fatty acid profile, total phenolic compounds (TPC), antioxidant capacity (AC), and spectral features using mid-infrared (MIR) and Raman spectroscopy. The results revealed significant compositional differences among localities, particularly in fat, protein, and salt content, with Daniel Hernández milk showing higher nutritional density. The fatty acid profile, although statistically similar across districts, highlighted a favorable nutritional composition dominated by oleic, palmitic, and stearic acids. TPC and AC values were homogeneous among districts, reflecting similar feeding and management practices. Molecular vibration analysis via MIR and Raman spectroscopy allowed for the identification of key biochemical differences, particularly in lipid and carbohydrate regions. SIMCA classification models, based on MIR spectral data, successfully discriminated samples by origin with Inter-Class Distance (ICD) values exceeding 3, confirming statistically significant separation. Discriminating power plots revealed that proteins (amide I), lactose (C–O, C–C), and lipid-associated bands (C=O, CH_2_) were major contributors to class differentiation. These findings demonstrate the effectiveness of combining spectroscopic and chemometric approaches to trace the geographical origin of milk and provide scientific support for potential quality labeling systems. This methodology contributes to ensuring product authenticity, promoting regional value-added dairy production, and supporting sustainable rural development in high-Andean ecosystems.

## 1. Introduction

Bovine milk, a liquid secreted by the mammary glands of mammals, has long been considered one of the most complete foods due to its richness in essential nutrients such as proteins, fats, vitamins, and minerals [1]. The composition of bovine milk is influenced by multiple factors, including animal genetics, diet, production system, climate, altitude, and local environmental conditions [2,3]. In addition to its basic components (fat, protein, lactose, and mineral salts), milk contains bioactive substances such as peptides, oligosaccharides, and antioxidant compounds that play physiological roles beyond basic nutrition, including antimicrobial, antihypertensive, immunomodulatory, and antioxidant effects [4,5,6].

The physicochemical analysis of milk—including fat content, non-fat solids, protein, minerals, density, freezing point, pH, and electrical conductivity—provides key indicators not only of its nutritional value but also of its hygienic quality and the health status of the mammary gland [7,8]. For instance, variations in density are often associated with differences in total solids and protein content [9]. The freezing point is a particularly stable index in bovine milk and is used to detect adulterations, especially the addition of water [10,11].

In recent years, the use of spectroscopic technologies (such as near- and mid-infrared with Fourier-transform—FTIR—, fluorescence, nuclear magnetic resonance, Raman spectroscopy, and UV-Vis spectroscopy), in combination with multivariate statistical or chemometric tools (PCA, PLS-DA, OPLS-DA, neural networks), has enabled the discrimination of milk or dairy products based on geographic origin, feeding system, production method, animal species, and more [6,12]. Beyond dairy applications, FTIR and Raman spectroscopy have become increasingly valuable tools in bioanalytical and microbiological research, offering rapid, non-destructive, and label-free molecular fingerprinting of biological samples. FTIR spectroscopy, in particular, has been successfully applied in microbial and environmental microbiology to study biomolecular structures and cellular metabolism [13,14]. Similarly, Raman spectroscopy provides complementary structural information through inelastic light scattering, allowing for the identification of molecular vibrations characteristic of proteins, lipids, and nucleic acids [15,16]. The integration of both techniques, combined with chemometric modeling, has shown great potential for predicting compositional and authenticity parameters in complex biological matrices, including milk and dairy products [17]. For example, a study conducted in China used amino acid profiles measured by liquid chromatography to classify milk samples according to their geographical origin with high accuracy through OPLS-DA [6]. Another study showed that combining FTIR spectra with fatty acid profiles enhances the authentication of milk production systems, outperforming each method used individually [18].

In the Peruvian context, particularly in high-Andean regions such as Tayacaja (Pampas Valley), dairy production plays a vital role in the local economy. In Peru, milk production reached approximately 2.2 million tons in 2024 (≈2.2 billion liters), according to the Ministry of Agricultural Development and Irrigation [19]. In the Pampas Valley, a study reported an average production of 3697 L per day among 77 producers, of which 44.8% was used for the artisanal production of dairy products such as cheese, yogurt, and milk caramel spread. These products are sold in local stores, street stalls, and through collectors who distribute them to Huancayo and Lima, major cities in Peru [20]. However, there is limited knowledge regarding the variability of bioactive components (especially total phenolic content and antioxidant capacity) and the application of spectroscopic fingerprinting and chemometric methodologies to distinguish milk samples by district, altitude, feeding practices, or environmental conditions. Such differentiation could contribute not only to improving milk quality and safety but also to promoting traceability and adding value to local dairy products.

Therefore, the objective of this study is to comprehensively characterize cow’s milk from different districts within the Pampas Valley (Tayacaja, Peru) by analyzing its physicochemical composition, bioactive properties, and spectroscopic fingerprint, and to assess the ability of chemometric tools to discriminate its geographical origin. Through this multidimensional approach, we aim to provide scientific evidence that supports quality enhancement strategies, territorial authenticity, and value-added initiatives for regional dairy production.

## 2. Results and Discussion

### 2.1. Physicochemical Characterization of Milk

The characterization of milk collected from different localities in the Pampas Valley (Table 1) showed notable variations in several components, reflecting differences in livestock management and environmental conditions. Fat content ranged from 2.385% in Daniel Hernández to 3.794% in Acraquia, while non-fat solids were more homogeneous, with values between 8.611% and 9.075%. This stability in non-fat solids aligns with previous studies indicating that, unlike milk fat, these components tend to remain constant despite differences in diet and stage of lactation [21]. Milk density showed significant variation, being highest in Daniel Hernández (1032.267 kg/m^3^), which is associated with a higher content of proteins and salts. This relationship between density and total solids is well-documented, indicating that milk with higher density reflects a richer nutrient composition [9]. Lactose levels showed slight differences (4.724–4.976%), remaining within the expected range and suggesting adequate glucose-lactose metabolism in the cows [2]. This is consistent with previous reports showing that lactose concentration in milk tends to remain relatively stable, although it may slightly increase in diets formulated with high energy content (cereal grains, maize silage, concentrate mixes of molasses, soybean meal, and by-products of grain processing) [22]. Protein and salt concentrations were highest in Daniel Hernández (3.326% and 0.743%, respectively). Studies by Poulsen et al. [23] and Ayers et al. [24] indicate that protein-enriched diets directly increase the protein and mineral content in milk, which could explain these findings. Such diets typically include high-protein feed ingredients such as soybean meal, rapeseed meal, or legume forages (e.g., alfalfa or clover), which enhance amino acid availability for milk protein synthesis.

In terms of physicochemical properties, the freezing point remained stable at around −0.55 °C in all samples, with no significant differences between locations. This result is consistent with the literature, which indicates that the freezing point is one of the most stable indices in cow’s milk and can be used as an indicator to detect adulteration with water [10,11]. The pH (≈6.0) and electrical conductivity (~5.2 mS/cm) values were also similar across all locations, reflecting a homogeneous composition and consistent post-milking management. Previous studies have shown that both pH and conductivity remain within ranges characteristic of normal milk [8,25]. In summary, although the Daniel Hernández location had milk with higher density and higher fat, protein, and salt content, the basic physicochemical parameters (pH, conductivity, and freezing point) showed remarkable uniformity among the samples, suggesting relatively stable production and management conditions throughout the Pampas Valley.

### 2.2. Bioactive Compounds

#### 2.2.1. Fatty Acids

The fatty acid profile (Table 2) was analyzed as part of the general physicochemical characterization and as an evaluation of bioactive compounds, given the well-known health effects of oleic, palmitoleic, and linoleic acids. No statistically significant differences (*p* < 0.05) were found among the four districts for any of the fatty acids identified by gas chromatography. Therefore, the average of all reported data (*n* = 5 samples per district) was used to represent the fatty acid content of bovine milk in the Pampas Valley. However, the absence of statistical differences may be attributed to the limited number of samples collected from each district’s farms, which hindered the ability to determine geographical discrimination based on fatty acids. On the other hand, the use of chemometric tools in combination with vibrational spectroscopy techniques, which typically involve a larger sample size, can reveal such differences [26].

The results shown in Table 2 indicate that short and medium chain fatty acids (C6:0 to C12:0), such as hexanoic (0.746%), octanoic (0.605%), decanoic (1.509%), and lauric (1.818%), are primarily synthesized de novo in the bovine mammary gland [27]. This is consistent with the findings of Ceciliani et al. [28] and Wang et al. [29], who reported similar concentrations in milk from grazing cows. Additionally, Poonia et al. [30] described the antimicrobial functions of these fatty acids, contributing to the immunological capacity of colostrum. Myristic acid (C14:0, 8.676%) was significantly higher than lauric acid (*p* < 0.05), as also observed by Palmquist et al. [27]. Its presence is relevant due to its ability to increase LDL cholesterol levels in humans, a finding that aligns with studies in human nutrition. In contrast, its unsaturated isomer, myristoleic acid (C14:1, 0.318%), is attributed to Δ9-desaturase activity [31]. Regarding long-chain saturated fatty acids, palmitic (C16:0, 27.581%) and stearic (C18:0, 16.379%) together represent over 44% of the total, aligning with values reported by Mensink et al. [32], who highlighted the C16:0/C18:0 ratio as a key factor in their effect on plasma cholesterol. Indeed, Mensink et al. [32] demonstrated that C18:0 does not negatively affect LDL levels, reducing cardiovascular risk compared to other saturated fats. Oleic acid (C18:1 cis, 31.710%), the principal monounsaturated fatty acid, is a typical marker of forage-based diets, consistent with findings by Lock & Bauman [33]. Similarly, Mozaffarian et al. [34] noted that this fatty acid improves the HDL/LDL ratio, offering cardioprotective benefits. Palmitoleic acid (C16:1, 0.586%) also supports the presence of Δ9-desaturase activity in the mammary gland, as proposed by Mosley & McGuire [35].

The trans-fatty acid fraction, represented by elaidic acid (C18:1 trans, 3.295%), may derive from vaccenic acid synthesized in the rumen. Lock & Bauman [33] warned that these trans isomers are associated with adverse metabolic effects, although the concentration found in this study is moderate, suggesting a balance between rumen efficiency and nutritional quality. Finally, linoleic acid (C18:2, 1.394%) is consistent with concentrations found in pasture-based systems, which promote the intake of omega-6 sources [36]. As a precursor of conjugated linoleic acid (CLA), its significance lies in its potential anti-inflammatory and anticancer effects as reported by Ou et al. [37]. The C16:0/C18:0 ratio, along with the presence of moderate amounts of monounsaturated and trans fats, aligns with relevant literature [38]. The nutritional quality of this composition is considered favorable, offering health benefits due to its high oleic and linoleic acid content and limited presence of trans fats [34,39]. This profile suggests that milk produced in the Pampas Valley has a notable functional and nutritional value compared to milk from less pasture-based production systems.

#### 2.2.2. Total Phenolic Compounds and Antioxidant Capacity

Table 3 shows that the average values of total phenolic compounds (TPC) ranged from 2.032 to 2.338 mg GAE/100 g, while antioxidant capacity (AC) ranged from 5.927 to 6.020 µmol Trolox/g. ANOVA analysis indicated that there were no significant differences among the four sampled locations in the Pampas Valley, as all shared the same letter in both variables (*p* < 0.05). This reflects a relative homogeneity in the composition of cow’s milk in the study area, possibly related to similar feeding and management conditions among the districts of the Pampas Valley [40].

In contrast, TPC values reported in other studies for bovine milk show notable variations due to methodological differences and result expression. For example, Vázquez et al. [41] reported values close to 49 mg GAE/L in bovine milk, which is approximately equivalent to 4.9 mg GAE/100 g, using a specific extraction protocol. These discrepancies with the values obtained in the Pampas Valley (≈2.0 mg GAE/100 g) may be attributed to differences in the extraction technique, sample preparation, and units used. Therefore, it is essential to consider methodology before making strict comparisons across studies [41]. Total phenolic compounds in bovine milk largely depend on the animal’s diet and ruminal metabolism, as well as individual microbiota and physiological factors. It has been demonstrated that forage-based feeding regimens or those supplemented with plant-based inputs modify the phenolic profile of milk and contribute to its antioxidant capacity [40,42].

In the present study, dairy cows sampled from the Pampas Valley (Tayacaja) were managed under small-scale Andean grazing systems, seasonally supplemented with locally harvested native pastures and limited concentrated feeds. The predominance of native forage in the diet is consistent with evidence showing higher levels of forage-derived phenolic metabolites (e.g., hippuric acid, enterolactone) in milk from grazing systems compared to intensive concentrate-based regimes [40,43]. Consequently, the predominantly pasture- and mixed forage-based diet practiced in the region is likely to influence the phenolic composition and associated antioxidant capacity of the milk samples analyzed.

The homogeneity observed in the samples from the Pampas Valley could indicate that cows are receiving a similar feeding regimen, based mainly on local grazing. Although the Folin–Ciocalteu method is widely used and reliable for estimating total phenolic compounds, it can also react with other molecules with reducing capacity [44]. Therefore, the values obtained primarily reflect the total phenolic fraction and should be interpreted with this in mind. For future studies, it is recommended to complement these determinations with chromatographic techniques (HPLC or LC–MS) to identify individual phenols, as well as to use additional antioxidant assays such as ABTS (2,2′-Azino-bis (3-ethylbenzothiazoline-6-sulfonic acid)), FRAP (Ferric Reducing Antioxidant Power), or ORAC (Oxygen Radical Absorbance Capacity), which would allow for a more comprehensive characterization of the milk’s antioxidant potential.

### 2.3. Molecular Vibrations

#### 2.3.1. Mid-Infrared (MIR) Spectroscopy Analysis

**Figure 1:**
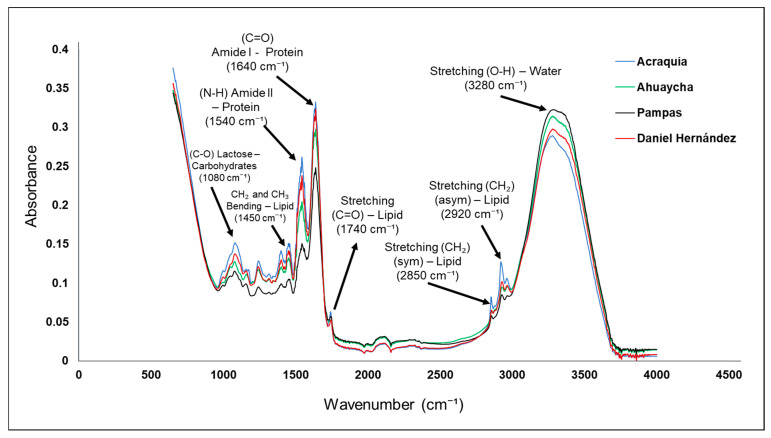
Infrared vibrational spectra of cow milk from the Pampas Valley districts.

Fourier-transform infrared (FTIR) spectroscopy applied to bovine milk from the districts of Pampas, Acraquia, Ahuaycha, and Daniel Hernández enabled the identification of apparent differences in molecular composition that may reflect the environmental conditions and livestock practices specific to each district (Figure 1). This technique proves effective for non-destructive analysis of key biochemical constituents in dairy foods (such as proteins, lipids, and carbohydrates) through the evaluation of specific molecular vibrations [45]. In the region between 3200 and 3400 cm^−1^, a broad band is observed, attributed to the stretching of O–H bonds, which largely reflect water content and, to a lesser extent, N–H bonds in proteins [46]. The differences in this band among samples may be related to variations in the water/total solids balance.

The milk from the Daniel Hernández district shows a slightly higher intensity in this region, which could indicate higher water content or a more intense interaction with proteins. Similarly, the bands recorded between 2850 and 2950 cm^−1^ correspond to the symmetric and asymmetric stretching of methylene (CH_2_) and methyl (CH_3_) groups, present in the aliphatic chains of lipids [47]. The greater intensity of these bands in the Ahuaycha sample suggests a higher fat concentration, which could be due to a different feeding regime, possibly richer in seeds or fatty pastures [48,49]. This observation is consistent with studies showing that cattle diet directly affects the lipid profile detectable by infrared spectroscopy [43]. The region between 1600 and 1650 cm^−1^ exhibits the so-called amide I band, characteristic of proteins, particularly caseins, which are fundamental to the nutritional and functional quality of milk. This band arises from the C=O stretching of the peptide bond [50]. The intensities observed in the analyzed samples indicate a relatively homogeneous protein content across the zones, although Acraquia shows a slight superiority in this signal, which may be linked to a more traditional livestock management based on high-altitude natural pastures [48,51,52]. On the other hand, the region from 1000 to 1100 cm^−1^ is associated with C–O and C–C stretching vibrations of carbohydrates—especially lactose, the main sugar in [53,54]. The Daniel Hernández sample exhibits higher intensity in this region, suggesting a slightly higher lactose content (Table 1), possibly resulting from the use of energy concentrate supplementation, a practice more common in more technical livestock systems. The spectral differences observed not only enable evaluation of the compositional quality of the samples, but also suggest potential indicators for geographic traceability and authenticity of dairy products. Previous studies have highlighted the usefulness of FTIR spectroscopy in detecting adulterations, dilutions, and structural changes resulting from milk manipulation, making it a valuable tool in quality assurance and origin differentiation [55].

Finally, it is important to emphasize that milk obtained in high-Andean contexts, such as those studied, tends to exhibit spectral profiles rich in protein and lipid components, partly as an adaptive response of cattle to altitude conditions, where metabolism is optimized to produce more concentrated milk [56,57]. These features may be leveraged in the development of value-added products, such as artisanal cheeses or functional milks, whose differentiation can be scientifically supported via spectroscopic analyses like the present one.

#### 2.3.2. Raman Spectroscopy Analysis

**Figure 2:**
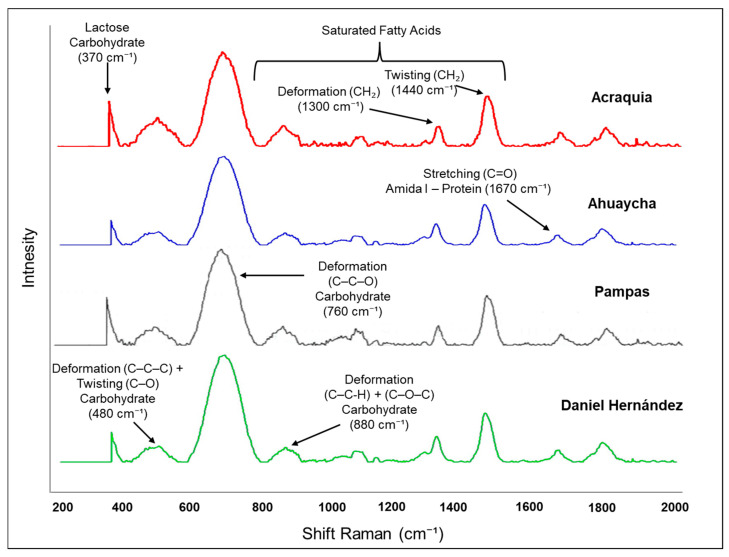
Raman spectra of cow’s milk from the Pampas Valley districts.

In the Raman spectra of bovine milk from the four districts of the Pampas Valley (Figure 2), several representative bands can be distinguished. One of the most notable findings is the presence of bands attributed to carbohydrates, particularly lactose, observed in the region between 800 and 1200 cm^−1^. According to Almeida et al. [58], these regions are associated with vibrations of C–C, C–O bonds and ether groups (C–O–C) typical of milk sugars, and are indicative of lactose content and its interaction with other components such as whey and proteins. Protein-related bands, especially those corresponding to the amide I group (~1670 cm^−1^), were particularly evident in all districts. As noted by Khan et al. [59], the amide I band is associated with the stretching of the C=O bond in peptides and has been extensively used to assess both the quantity and secondary structure of milk proteins. Regarding lipids, characteristic bands of methylene groups (CH_2_) were identified in the region between 1300 and 1440 cm^−1^, corresponding to deformations and torsions of C–H bonds, which are representative of the milk fat content [60]. It is important to note that these spectral regions allow for the discrimination between milk samples from different origins or treatments, highlighting the sensitivity of Raman spectroscopy in detecting subtle variations in the milk matrix [61].

Regarding the intense band near 760 cm^−1^, it can be attributed to deformation vibrations of C–C–C and C–C–O bonds, which are typical of carbohydrates and phospholipids present in the milk fat globule membrane [45,62]. Likewise, a weaker band appears around 1800 cm^−1^, suggesting the presence of C=O stretching vibrations corresponding to lipid esters or free fatty acids/minor carbonyl compounds [62]. These bands are absent or very weak in other studies on raw milk or milk powder [45,63], which can be explained by differences in the milk matrix, phospholipid/lipid content, Raman acquisition conditions, or the degree of sample homogenization. The presence of these bands in our samples indicates that the Raman spectrum is sensitive to contributions from lipid membranes and minor fatty acids in milk produced in high-Andean regions.

Raman spectroscopy has proven to be useful in identifying compositional differences in dairy products due to its ability to detect variations in band intensities associated with lipids, proteins, and carbohydrates. Although multivariate classification analyses were not applied in this study, their future implementation is recommended to deepen the classification of samples according to their district of origin, allowing for the identification of spectral groupings and potential quality markers [60]. However, it is important to acknowledge certain limitations of this technique. Band overlap, natural fluorescence of some compounds, and sensitivity to experimental conditions can hinder accurate spectral interpretation [58,59].

### 2.4. Chemometric Analysis

Based on the spectral data obtained through mid-infrared (MIR) spectroscopy, SIMCA classification analysis was applied to bovine milk samples collected from four districts within the province of Tayacaja, located in the department of Huancavelica, Peru. This statistical approach relies on the creation of independent models for each class (or district, in this case), using Principal Component Analysis (PCA) as a preliminary step. PCA reduces the dimensionality of spectral data while preserving most of the original variance. In SIMCA projection plots, class separation is evaluated using the Inter-Class Distance (ICD), a metric that quantifies the multivariate distance between the class models generated for each group. An ICD value greater than 3 is considered statistically significant, indicating a clear differentiation between the analyzed classes [46].

**Figure 3:**
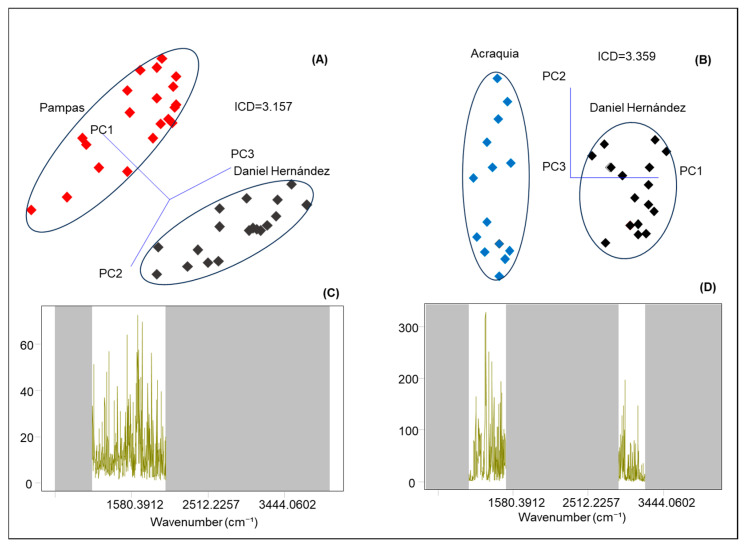
SIMCA projection scores plots (**A**,**B**) and discriminating power (**C**,**D**) in cow’s milk from the districts of Pampas, Daniel Hernández, and Acraquia using a portable FTIR system.

Figure 3A presents a comparison between samples from Pampas (shown in red) and Daniel Hernández (shown in black). In this case, the analysis yields an ICD of 3.16, suggesting that the physicochemical characteristics of the milk from these two localities exhibit substantial differences. This separation indicates that the infrared spectral profiles—driven by milk composition—are sufficiently distinct to allow for reliable classification. Furthermore, the discriminating power plot (Figure 3C) identifies the main bands responsible for these differences between districts, which are associated with proteins (amide I: 1600–1650 cm^−1^), lactose (C–O and C–C: 1000–1100 cm^−1^), and to a lesser extent, the lipid fraction (C=O: 1730 cm^−1^). On the other hand, Figure 3B shows the comparison between samples from Acraquia and Daniel Hernández, with an even higher ICD value of 3.40. In Figure 3D, the discriminating power plot again highlights protein-related bands (amide I: 1600–1650 cm^−1^) and lactose (C–O and C–C: 1000–1100 cm^−1^), along with strongly pronounced lipid-associated bands (symmetric CH_2_ stretching: 2850 cm^−1^ and asymmetric: 2918 cm^−1^). It is worth noting that in both cases, the samples are clearly grouped within ellipses that delineate each class (Figure 3A,B), demonstrating the high reliability of the SIMCA model in correctly assigning samples based on their geographic origin. The observed differences can be attributed to various agroecological factors that vary between locations, such as altitude, type of pasture consumed by cattle, water availability and quality, as well as livestock management practices in each area. All of these factors directly influence milk composition, both at the macronutrient level and in its metabolomic profile [43,55,60,61].

In summary, the results obtained demonstrate that the combined application of infrared spectroscopy and multivariate statistical analyses such as SIMCA is effective for the geographical traceability of bovine milk. This discriminative capacity is not only useful in the context of product quality control and authenticity, but also has significant implications for the valorization of regional products. In a region like Tayacaja, characterized by its ecological and cultural diversity, these tools could be fundamental in establishing systems of origin denomination, promoting differentiated quality certifications, and thereby supporting the sustainable economic development of rural communities dedicated to dairy production.

## 3. Materials and Methods

### 3.1. Raw Material

A total of 120 samples (30 per district in the Pampas Valley) of raw milk (Brown Swiss cattle breed), each with a volume of 500 mL, were collected from livestock producers located in the four representative districts of the Pampas Valley: Acraquia, Ahuaycha, Pampas, and Daniel Hernández. The samples were taken directly from the milking container after manual or mechanical milking, following standard hygienic conditions. To ensure representativeness, the milk was previously homogenized by manual agitation. Each sample was placed in metal bottles, previously identified and coded, and stored under refrigerated conditions at 4 °C in thermal boxes with cold accumulators. Transport to the laboratory was carried out within 4 h of collection to preserve the integrity of the physicochemical and microbiological parameters. Once in the laboratory, the samples were kept refrigerated until analysis, which was carried out within the first 24 h after collection. For the experimental design of the study, subsamples or fractions were obtained from the total number of samples collected (*n* = 120) for the different analyses performed in this study. Each milk sample was fractionated and processed according to the requirements of each analytical technique. The 120 complete samples were used for physicochemical characterization and infrared spectroscopy (MIR) analysis (40 per district). From this same set of samples, representative fractions were taken from a total of 40 samples for the determination of total phenolic compounds (TPC), antioxidant capacity (AC), and Raman spectroscopy (10 per district). Finally, 20 samples (5 per district) were selected from the original set for the determination of fatty acids.

### 3.2. Physicochemical Characteristics

The physicochemical characteristics of the milk, including fat content (%), non-fat solids (%), density (kg/m^3^), lactose (%), salts (%), protein (%), freezing point (°C), pH and conductivity (mS/cm), were determined using a Lactoscan SA50 (BOECKEL, serial number 033984, Nova Zagora, Bulgaria). For each analysis, 30 mL of milk sample was taken and placed in a 50 mL bottle. Subsequently, a measuring sensor (BOECKEL, model 224985, Nova Zagora, Bulgaria) was introduced directly onto the sample. After a stabilization time of 30 s, the equipment automatically reported all the above-mentioned parameters. Between each measurement, the electrode was subjected to a rigorous cleaning process using distilled water and alcohol to ensure complete removal of residues or impurities. Each sample was analyzed in triplicate to ensure the accuracy and repeatability of the data obtained.

### 3.3. Bioactive Compounds Determination

#### 3.3.1. Determination of Fatty Acids

Milk fat was extracted using the Gerber method. Ten milliliters of milk were measured with a pipette and transferred to a butyrometer, to which 10 mL of concentrated sulfuric acid and 1 mL of amyl alcohol were carefully added. The mixture was vigorously shaken until the proteins were completely dissolved. Subsequently, the butyrometers were centrifuged for 5 min in a Lacter 21 centrifuge (Fidel Pajares S.L., Madrid, Spain), previously thermostatted at 65 °C. The fatty acid determination was carried out following the AOAC (2005) fatty acid methyl ester method No. 991.39 [64]. For this purpose, 0.025 g of fat was weighed and reacted with 1.5 mL of 0.5 N NaOH at 90 °C in a water bath (Foos, model WB1024, Hillerød, Denmark) for 5 min. The mixture was then cooled to 30 °C, and 2.0 mL of boron trifluoride (BF_3_) was added, followed by heating again at 100 °C for 30 min. After cooling, 1 mL of isooctane and 5 mL of saturated NaCl solution were added, maintaining constant stirring under a nitrogen atmosphere to prevent oxidation. The separation and identification of fatty acids were performed using a gas chromatograph (GC) Shimadzu, model GC-2010 (Shimadzu Corporation, Kyoto, Japan), equipped with a flame ionization detector (FID) and an autosampler Shimadzu AOC-20Si (Shimadzu Corporation, Kyoto, Japan). A SP Rt™-2560 silica capillary column (100 m × 0.25 mm, 0.20 μm film thickness, Restek Corporation, Bellefonte, PA, USA) was used, with helium as the carrier gas at a flow rate of 30 mL/min and a pressure of 261.5 kPa. The injection volume was 1 μL, with an injector temperature set at 225 °C (split mode) and a detector temperature of 250 °C. The oven temperature program was: initial temperature of 100 °C held for 4 min, then ramped to 240 °C at 3 °C/min and held for 10 min.

#### 3.3.2. Determination of Total Phenolic Compounds (TPC)

The total phenolic content in the milk samples was determined using the colorimetric Folin–Ciocalteu method, adapted from Saura-Calixto et al. [65], with modifications for its application to dairy matrices. Firstly, the extraction of total phenolic compounds from the milk samples was carried out using solvent mixtures of methanol/water (50:50 *v*/*v*) and acetone/water (70:30 *v*/*v*), adjusted to pH 2 by the addition of 2N HCl. In 15 mL Falcon tubes, 5 mL of milk were mixed with 10 mL of the corresponding solvent. The mixtures were then subjected to ultrasonic bath treatment for 30 min to promote the breakdown of structures and the release of phenolic compounds. Subsequently, the samples were centrifuged using a Sigma centrifuge (model 2-16P, Osterode am Harz, Germany) at 3000 rpm for 20 min. The resulting supernatant was carefully collected and stored at 5 °C until analysis. Afterward, a standard solution of gallic acid (C_7_H_6_O_5_) at a concentration of 450 μg/mL was prepared by dissolving 0.0225 g of the compound in 50 mL of distilled water. Additionally, a 20% (*w*/*v*) sodium carbonate (Na_2_CO_3_) solution was prepared by dissolving 2 g of the reagent in 10 mL of distilled water, followed by sonication for 5 min to ensure homogeneous dissolution. The Folin–Ciocalteu reagent was used in its 2N form, according to the manufacturer’s instructions. From the stock solution of gallic acid, standard solutions were prepared at concentrations of 7.2, 14.4, 21.6, 28.8, and 36.0 μg/mL, which were used to construct the calibration curve. In Eppendorf tubes, 20, 40, 60, 80, and 100 μL of each respective concentration were placed, followed by the addition of 100 μL of the 2N Folin–Ciocalteu reagent. These mixtures were left to stand for 5 min at room temperature. Subsequently, 50 μL of the 20% sodium carbonate solution were added, and distilled water was used to bring the final volume of each tube to 1.2 mL. After a 1 h incubation in the dark, 200 μL of each sample were transferred to a microplate, and absorbance was measured at a wavelength of 730 nm using a Synergy™ H1 multimode microplate reader (BioTek Instruments, Inc., Cheadle, UK). Based on the absorbance readings, a calibration curve was constructed, yielding the equation Y = 0.0455X − 0.044, with a determination coefficient of R^2^ = 0.997, where X represents the gallic acid concentration (mg GAE/mL) and Y the measured absorbance.

#### 3.3.3. Antioxidant Capacity (AC)

The antioxidant capacity of the milk samples was determined using the DPPH free radical method, adapted from the procedure described by Kim et al. [66], with specific modifications for its application to dairy matrices. For this purpose, a stock solution of Trolox at a concentration of 1 mM was prepared by dissolving 0.0125 g of the reagent in 50 mL of 80% methanol. Similarly, a DPPH solution (2,2-diphenyl-1-picrylhydrazyl) at a concentration of 1 mM was prepared by dissolving 3.9 mg of the reagent in 100 mL of 80% methanol. This solution was stored in an amber glass bottle to protect it from light and was homogenized for one hour using a magnetic stirrer (VELP Scientifica, ARE model, Usmate Velate, Italy). The DPPH solution was prepared immediately before use and kept refrigerated at 5 °C until analysis. From the Trolox stock solution, a series of standard concentrations (500, 400, 200, 100, 50, 25, 10, and 5 μM) were prepared to construct the calibration curve. For each standard concentration, 10 μL of the respective solution were mixed with 190 μL of the DPPH solution. The mixtures were allowed to react for 10 min at room temperature in darkness. Absorbance was then measured at a wavelength of 515 nm using a Synergy™ H1 multimode microplate reader (BioTek Instruments, Inc., Cheadle, UK). Based on the absorbance data, a calibration curve was constructed, resulting in the following equation: Y = 0.0503X + 6.5008, with a coefficient of determination R^2^ = 0.9965, where X represents the Trolox concentration (μmol/mL) and Y the percentage of DPPH radical inhibition. Once the calibration curve was established, the antioxidant capacity of the milk samples was analyzed. For each sample (previously extracted and conditioned), 10 μL were placed in a microplate well, followed by the addition of 190 μL of the DPPH solution. The mixtures were incubated for 10 min in the dark at room temperature. Subsequently, absorbance was measured at 515 nm using the same multimode reader. Each milk sample was analyzed in triplicate to ensure reproducibility and accuracy of the results. Finally, the antioxidant capacity of the milk samples was calculated using the Trolox calibration curve, and the results were expressed as micromoles of Trolox equivalents per milliliter of milk (μmol Trolox/mL).

### 3.4. Infrared Spectroscopy

Milk samples were analyzed using an approximate volume of 0.2 mL with the help of a portable mid-infrared (MIR) spectrometer (Agilent Technologies, Cary 630 FTIR, Santa Clara, CA, USA), operating in the absorption band range of 650 to 4000 cm^−1^. The device was equipped with a triple-reflection diamond attenuated total reflectance (ATR) accessory. Spectral acquisition was programmed with 64 scans at a resolution of 4 cm^−1^. The milk samples were placed directly on the ATR surface after background correction. The ATR surface was carefully cleaned after each measurement using 95% acetone and 99% ethanol, followed by wiping with tissue paper to prevent contamination. All samples were measured in duplicate to ensure data accuracy. The spectral data were collected and processed using Microlab software (Agilent Technologies, version 5.8, Waldbronn, Germany).

### 3.5. Raman Spectroscopy

The collection of Raman spectra was carried out using quartz cuvettes with a capacity of 1.5 mL. The analysis was performed using a handheld Rigaku Progeny, part of the Progeny series, Raman spectrometer (Rigaku Corporation, Akishima, Tokyo, Japan), which operates with a 1064 nm excitation laser and is equipped with an InGaAs detector (indium gallium arsenide array, Rigaku Corporation, Akishima, Tokyo, Japan). This configuration minimizes fluorescence interference, delivering high sensitivity in the detection of organic compounds. For each sample, the laser was set to an output power of 450 mW, with 15 scans and an exposure time of 1200 milliseconds per scan. The Raman shift range considered was 200 to 2000 cm^−1^, enabling the acquisition of detailed vibrational information from the components present in the milk. Spectral acquisition was performed using the Progeny software (Rigaku Corporation, version 1.7.5.x, Akishima, Tokyo, Japan), and each sample was analyzed in duplicate to ensure data reproducibility. To prevent cross-contamination between measurements, the quartz cuvettes were thoroughly cleaned with 95% acetone after each use.

### 3.6. Statistical Analysis

A completely randomized design (CRD) was used to compare treatment means through analysis of variance (ANOVA) at a 95% confidence level (*p* < 0.05). Significant differences among means were determined using Tukey’s test, with the support of the statistical software Minitab^®^ (version 18; Minitab LLC, State College, PA, USA). All analyses related to the physicochemical characterization and bioactive components were performed in triplicate, in order to ensure the reproducibility and reliability of the results. For the discrimination of cattle milk between localities in the Pampas Valley, the soft independent model of class analogy (SIMCA) was used for supervised classification based on principal component analysis (PCA), using multivariate statistical analysis software (Pirouette^®^ version 4.0, Infometrix Inc., Woodville, WA, USA). The SIMCA discriminant power graph identified the main infrared bands associated with the sample classifications [47]. The visualization of the sample grouping was achieved by projecting the score plot of the original data onto the PCA axes. Inter-Class Distance (ICD) greater than three (ICD > 3) determined a significant discrimination between milk samples from the Pampas Valley localities.

## 4. Conclusions

The physicochemical, bioactive, and spectroscopic characterization study of milk produced in the Pampas Valley demonstrates the influence of agroecological and livestock management conditions on its composition and quality. The results show that, although variations exist among districts, particularly in fat, protein, mineral, and density content. The basic parameters such as pH, freezing point, and conductivity remained stable, reflecting consistent post-milking management practices. Milk from the district of Daniel Hernández stood out for its higher density and protein content, suggesting diets with greater nutrient contributions. In the fatty acid profile across all districts of the Pampas Valley, medium- and long-chain fatty acids predominated, with a high proportion of oleic acid and a favorable balance between saturated and monounsaturated fatty acids, highlighting the nutritional and functional value of milk from the Pampas Valley. The moderate presence of trans fatty acids and the appropriate palmitic/stearic acid ratio further reinforce its healthy character. Similarly, the levels of phenolic compounds and antioxidant capacity remained uniform among the districts of the Pampas Valley. Spectroscopic analyses using FTIR and Raman techniques allowed for the identification of molecular variations associated with proteins, lipids, and carbohydrates. The application of the chemometric SIMCA model to the FTIR data confirmed a clear differentiation among bovine milk samples from certain districts of the Pampas Valley, with significant interclass distances (ICD > 3), validating the discriminant capacity of the spectral profiles. Overall, the results reveal that milk from the Pampas Valley possesses a balanced composition, with outstanding nutritional attributes and potential for valorization through modern analytical tools, providing a solid foundation for the development of differentiated dairy products and denomination of origin strategies that promote regional sustainable development.

## Figures and Tables

**Table 1:** Characterization of milk from the Pampas Valley.

Component	Acraquia	Ahuaycha	Pampas	Daniel Hernández	Pampas Valley *
Fat (%)	3.794 ± 0.342 ^a^	3.778 ± 0.822 ^a^	2.920 ± 0.075 ^ab^	2.385 ± 0.789 ^b^	3.219 ± 0.714
Non-Fat Solids (%)	8.611 ± 0.929 ^b^	8.766 ± 0.596 ^a^	8.719 ± 0.398 ^ab^	9.075 ± 0.376 ^a^	8.793 ± 0.632
Density (kg/m^3^)	1029.200 ± 5.063 ^b^	1029.877 ± 3.609 ^b^	1030.452 ± 1.960 ^ab^	1032.267 ± 1.801 ^a^	1030.460 ± 3.526
Lactose (%)	4.724 ± 0.519 ^b^	4.809 ± 0.334 ^ab^	4.787 ± 0.220 ^ab^	4.976 ± 0.221 ^a^	4.824 ± 0.354
Salts (%)	0.704 ± 0.078 ^b^	0.716 ± 0.050 ^ab^	0.712 ± 0.033 ^ab^	0.743 ± 0.032 ^a^	0.719 ± 0.053
Protein (%)	3.146 ± 0.353 ^b^	3.202 ± 0.230 ^ab^	3.191 ± 0.149 ^ab^	3.326 ± 0.141 ^a^	3.216 ± 0.241
Freezing Point (°C)	−0.550 ± 0.058 ^a^	−0.560 ± 0.035 ^a^	−0.552 ± 0.027 ^a^	−0.574 ± 0.025 ^a^	−0.559 ± 0.039
pH	6.063 ± 0.398 ^a^	5.914 ± 1.006 ^a^	6.213 ± 0.613 ^a^	5.977 ± 0.491 ^a^	6.042 ± 0.669
Conductivity (mS/cm)	5.026 ± 0.580 ^a^	5.134 ± 0.568 ^a^	5.245 ± 0.595 ^a^	5.323 ± 0.462 ^a^	5.182 ± 0.558

Equal letters in the same row do not show significant differences (*p* < 0.05). * Values in this column represent the average across the four sampled districts of the Pampas Valley.

**Table 2:** Quantification of fatty acids in milk from the Pampas Valley.

Fatty Acids	Concentration (%)
Hexanoic acid (C6:0)	0.746 ± 0.011 ^g^
Octanoic acid (C8:0)	0.605 ± 0.002 ^g^
Decanoic acid (C10:0)	1.508 ± 0.010 ^f^
Lauric acid (C12:0)	1.818 ± 0.015 ^f^
Myristic acid (C14:0)	8.676 ± 0.172 ^d^
Myristoleic acid (C14:1)	0.319 ± 0.001 ^h^
Palmitic acid (C16:0)	27.582 ± 1.623 ^b^
Palmitoleic acid (C16:1)	0.586 ± 0.003 ^g^
Stearic acid (C18:0)	16.379 ± 1.525 ^c^
Oleic acid (C18:1)	31.710 ± 1.820 ^a^
Elaidic acid (C18:1 (trans))	3.294 ± 0.250 ^e^
Linoleic acid (C18:2)	1.394 ± 0.023 ^f^

Equal letters in the same row do not show significant differences (*p* < 0.05).

**Table 3:** Quantification of total phenolic compounds (TPC) and antioxidant capacity (AC) in milk from cows in the Pampas Valley.

Component	Acraquia	Ahuaycha	Pampas	Daniel Hernández	Pampas Valley *
TPC (mg GAE/100 g)	2.031 ± 0.020 ^a^	2.335 ± 0.031 ^a^	2.338 ± 0.016 ^a^	2.024 ± 0.025 ^a^	2.182 ± 0.025
AC (µmol Trolox/g)	5.926 ± 0.048 ^a^	5.990 ± 0.034 ^a^	6.022 ± 0.020 ^a^	5.950 ± 0.054 ^a^	5.970 ± 0.044

Equal letters in the same row do not show significant differences (*p* < 0.05). * Values in this column represent the average across the four sampled districts of the Pampas Valley.

## Data Availability

All research data are contained in the main text.

## References

[1] Chalupa-Krebzdak S., Long C.J., Bohrer B.M. (2018). Nutrient density and nutritional value of milk and plant-based milk alternatives. Int. Dairy J..

[2] Fox P.F., McSweeney P.L.H. (2003). Advanced Dairy Chemistry—1 Proteins.

[3] McPherson S., Vasseur E. (2020). Graduate Student Literature Review: The effects of bedding, stall length, and manger wall height on common outcome measures of dairy cow welfare in stall-based housing systems. J. Dairy Sci..

[4] Korhonen H. (2009). Milk-derived bioactive peptides: From science to applications. J. Funct. Foods.

[5] Hsieh C., Hernández-Ledesma B., Fernández-Tomé S., Weinborn V., Barile D., De Moura Bell J.M.L.N. (2015). Milk Proteins, Peptides, and Oligosaccharides: Effects against the 21st Century Disorders. BioMed Res. Int..

[6] Kang M., Yue Q., Jia S., Wang J., Zheng M., Suo R. (2022). Identification of Geographical Origin of Milk by Amino Acid Profile Coupled with Chemometric Analysis. J. Food Qual..

[7] Otwinowska-Mindur A., Ptak E. (2018). Effects of lactation number, milk yield and milk composition on freezing point of milk of Polish Holstein-Friesian cows. J. Cent. Eur. Agric..

[8] Sharma A., Sharma S., Singh N., Poonia M.P. (2016). Evaluation of electric conductivity and milk lactose as a pre-indicator of mastitis in Tharparkar dairy cattle. Indian J. Anim. Prod. Manag..

[9] Parmar P., Lopez-Villalobos N., Tobin J.T., Murphy E., McDonagh A., Crowley S.V., Kelly A.L., Shalloo L. (2020). The Effect of Compositional Changes Due to Seasonal Variation on Milk Density and the Determination of Season-Based Density Conversion Factors for Use in the Dairy Industry. Foods.

[10] Pindešová I.F., Fehér A., Prus P., Zajác P., Prčík M. (2022). Farm Level Milk Adulteration: Changes in the Physicochemical Properties of Raw Cow’s Milk after the Addition of Water and NaCl. Agriculture.

[11] Pesce A., Salzano C., De Felice A., Garofalo F., Liguori S., De Santo A., Palermo P., Guarino A. (2016). Monitoring the freezing point of buffalo milk. Ital. J. Food Saf..

[12] Xu L., Deng D.H., Cai C.B., Yang H.W. (2011). Automatic discrimination of the geographical origins of milks by excitation-emission fluorescence spectrometry and chemometrics. J. Autom. Methods Manag. Chem..

[13] Kassem A., Abbas L., Coutinho O., Opara S., Najaf H., Kasperek D., Pokhrel K., Li X., Tiquia-Arashiro S. (2023). Applications of Fourier Transform-Infrared Spectroscopy in Microbial Cell Biology and Environmental Microbiology: Advances, Challenges, and Future Perspectives. Front. Microbiol..

[14] Kamnev A.A., Tugarova A.V. (2023). Specificities of the Fourier Transform Infrared Spectroscopic Methodology and Interpretation of Spectroscopic Data in Microbiological Analyses. J. Anal. Chem..

[15] Pezzotti G. (2021). Raman Spectroscopy in Cell Biology and Microbiology. J. Raman Spectrosc..

[16] Vulchi R.T., Morgunov V., Junjuri R., Bocklitz T. (2024). Artifacts and Anomalies in Raman Spectroscopy: A Review on Origins and Correction Procedures. Molecules.

[17] Mohammadi S., Gowen A., Luo J., O’Donnell C. (2024). Prediction of Milk Composition Using Multivariate Chemometric Modelling of Infrared, Raman, and Fluorescence Spectroscopic Data: A Review. Food Control.

[18] Bergamaschi M., Cipolat-Gotet C., Cecchinato A., Schiavon S., Bittante G. (2020). Chemometric authentication of farming systems of origin of food (milk and ripened cheese) using infrared spectra, fatty acid profiles, flavor fingerprints, and sensory descriptions. Food Chem..

[19] Ministry of Agrarian Development and Irrigation (MIDAGRI) Peru’s Fresh Milk Production Reached 2.2 Million Tons in 2023. Agraria.pe. 3 June 2024. https://www.agraria.pe/noticias/produccion-de-leche-fresca-de-peru-alcanzo-las-2-2-millones--35938.

[20] Chamorro G. (2008). Comercialización de la leche y Derivados en el Valle de Pampas–Tayacaja. Bachelor’s Thesis.

[21] Dehghan-Banadaky M., Ebrahimi M., Motameny R., Heidari S. (2012). Effects of live yeast supplementation on mid-lactation dairy cows performances, milk composition, rumen digestion and plasma metabolites during hot season. J. Appl. Anim. Res..

[22] Slots T., Butler G., Leifert C., Kristensen T., Skibsted L., Nielsen J. (2009). Potentials to differentiate milk composition by different feeding strategies. J. Dairy Sci..

[23] Poulsen N., Giagnoni G., Johansen M., Lund P., Larsen L. (2021). Effect of protein concentrate mixtures and dietary addition of exogenous phytase on major milk minerals and proteins, including casein phosphorylation. J. Dairy Sci..

[24] Ayers A., Ziegler S., Darby H., Bosworth S., Alvez J., Colby J., Kraft J., Greenwood S. (2021). Assessment of dietary protein supplementation on milk productivity of commercial organic dairy farms during the grazing season. J. Dairy Sci..

[25] Stanek P., Żółkiewski P., Januś E. (2024). A Review on Mastitis in Dairy Cows Research: Current Status and Future Perspectives. Agriculture.

[26] Maurer N.E., Hatta-Sakoda B., Pascual-Chagman G., Rodriguez-Saona L.E. (2012). Characterization and authentication of a novel vegetable source of omega-3 fatty acids, sacha inchi (*Plukenetia volubilis* L.) oil. Food Chem..

[27] Palmquist D.L., Beaulieu A.D., Barbano D.M. (1993). Feed and Animal Factors Influencing Milk Fat Composition. J. Dairy Sci..

[28] Ceciliani F., Maggiolino A., Biscarini F., Dadi Y., De Matos L., Cremonesi P., Landi V., De Palo P., Lecchi C. (2024). Heat stress has divergent effects on the milk microbiota of Holstein and Brown Swiss cows. J. Dairy Sci..

[29] Wang F., Chen M., Luo R., Huang G., Wu X., Zheng N., Zhang Y., Wang J. (2021). Fatty acid profiles of milk from Holstein cows, Jersey cows, buffalos, yaks, humans, goats, camels, and donkeys based on gas chromatography–mass spectrometry. J. Dairy Sci..

[30] Poonia A., Shiva (2022). Bioactive compounds, nutritional profile and health benefits of colostrum: A review. Food Prod. Process. Nutr..

[31] Shingfield K., Toivonen V., Vanhatalo A., Huhtanen P., Griinari J. (2006). Short Communication: Indigestible Markers Reduce the Mammary Δ9-Desaturase Index and Alter the Milk Fatty Acid Composition in Cows. J. Dairy Sci..

[32] Mensink R.P., Zock P.L., Kester A.D., Katan M.B. (2003). Effects of dietary fatty acids and carbohydrates on the ratio of serum total to HDL cholesterol and on serum lipids and apolipoproteins: A meta-analysis of 60 controlled trials. Am. J. Clin. Nutr..

[33] Lock A.L., Bauman D.E. (2004). Modifying milk fat composition of dairy cows to enhance fatty acids beneficial to human health. Lipids.

[34] Mozaffarian D., Micha R., Wallace S. (2010). Effects on Coronary Heart Disease of Increasing Polyunsaturated Fat in Place of Saturated Fat: A Systematic Review and Meta-Analysis of Randomized Controlled Trials. PLoS Med..

[35] Mosley E.E., McGuire M.A. (2007). Methodology for the In Vivo Measurement of the Δ9-Desaturation of Myristic, Palmitic, and Stearic Acids in Lactating Dairy Cattle. Lipids.

[36] Loza C., Davis H., Malisch C., Taube F., Loges R., Magistrali A., Butler G. (2023). Ácidos grasos de la leche: El impacto del pastoreo de pastos diversos y el potencial para predecir el metano derivado del rumen. Agriculture.

[37] Ou L., Ip C., Lisafeld B., Ip M.M. (2007). Conjugated linoleic acid induces apoptosis of murine mammary tumor cells via Bcl-2 loss. Biochem. Biophys. Res. Commun..

[38] Wang L., Xu F., Song Z., Han D., Zhang J., Chen L., Na L. (2020). A high fat diet with a high C18:0/C16:0 ratio induced worse metabolic and transcriptomic profiles in C57BL/6 mice. Lipids Health Dis..

[39] Bauman D.E., Harvatine K.J., Lock A.L. (2011). Nutrigenomics, Rumen-Derived Bioactive Fatty Acids, and the Regulation of Milk Fat Synthesis. Annu. Rev. Nutr..

[40] Rocchetti G., Ghilardelli F., Mosconi M., Masoero F., Gallo A. (2022). Occurrence of Polyphenols, Isoflavonoids, and Their Metabolites in Milk Samples from Different Cow Feeding Regimens. Dairy.

[41] Vázquez C.V., Rojas M.G.V., Ramírez C.A., Chávez-Servín J.L., García-Gasca T., Martínez R.A.F., García O.P., Rosado J.L., López-Sabater C.M., Castellote A.I. (2014). Total phenolic compounds in milk from different species. Design of an extraction technique for quantification using the Folin–Ciocalteu method. Food Chem..

[42] Kuhnen S., Moacyr J.R., Mayer J.K., Navarro B.B., Trevisan R., Honorato L.A., Maraschin M., Filho L.C.P.M. (2014). Phenolic content and ferric reducing–antioxidant power of cow’s milk produced in different pasture-based production systems in southern Brazil. J. Sci. Food Agric..

[43] Fleming A., Schenkel F., Chen J., Malchiodi F., Bonfatti V., Ali R., Mallard B., Corredig M., Miglior F. (2017). Prediction of milk fatty acid content with mid-infrared spectroscopy in Canadian dairy cattle using differently distributed model development sets. J. Dairy Sci..

[44] Prior R.L., Wu X., Schaich K. (2005). Standardized Methods for the Determination of Antioxidant Capacity and Phenolics in Foods and Dietary Supplements. J. Agric. Food Chem..

[45] Mohammadi S., Gowen A., O’Donnell C. (2024). Vibrational spectroscopy data fusion for enhanced classification of different milk types. Heliyon.

[46] Rodriguez-Saona L., Ayvaz H., Ismail B.P., Nielsen S.S. (2024). Infrared and Raman Spectroscopy. Nielsen’s Food Analysis.

[47] Villanueva E., Glorio-Paulet P., Giusti M.M., Sigurdson G.T., Yao S., Rodríguez-Saona L.E. (2023). Screening for pesticide residues in cocoa (*Theobroma cacao* L.) by portable infrared spectroscopy. Talanta.

[48] Kostovska R., Cruz J., Drouin G., Horan B., Tobin J.T., O’Callaghan T.F., Hettinga K., Kelly A.L., Gómez-Mascaraque L.G. (2025). Use of Raman spectroscopy as a rapid tool to discriminate milk deriving from different pasture-based diets and breeds in a seasonal, spring-calving dairy production system. J. Dairy Sci..

[49] Gallardo W.B., Teixeira I.A.M.A. (2023). Associations between Dietary Fatty Acid Profile and Milk Fat Production and Fatty Acid Composition in Dairy Cows: A Meta-Analysis. Animals.

[50] Hussain R., Gaiani C., Aberkane L., Scher J. (2010). Characterization of high-milk-protein powders upon rehydration under various salt concentrations. J. Dairy Sci..

[51] Berhe T., Seifu E., Ipsen R., Kurtu M.Y., Hansen E.B. (2017). Processing Challenges and Opportunities of Camel Dairy Products. Int. J. Food Sci..

[52] O’Callaghan T.F., Hennessy D., McAuliffe S., Kilcawley K.N., O’Donovan M., Dillon P., Ross R., Stanton C. (2016). Effect of pasture versus indoor feeding systems on raw milk composition and quality over an entire lactation. J. Dairy Sci..

[53] Wiercigroch E., Szafraniec E., Czamara K., Pacia M.Z., Majzner K., Kochan K., Kaczor A., Baranska M., Malek K. (2017). Raman and infrared spectroscopy of carbohydrates: A review. Spectrochim. Acta A Mol. Biomol. Spectrosc..

[54] Gafour H.M., Bouterfas M. (2011). Harmonic Dynamics of α-D-Lactose in the Crystalline State. J. Mol. Imaging Dyn..

[55] Saji R., Ramani A., Gandhi K., Seth R., Sharma R. (2024). Application of FTIR spectroscopy in dairy products: A systematic review. Food Humanit..

[56] Alrhmoun M., Zanon T., Katzenberger K., Holighaus L., Gauly M. (2023). Exploring the heights: Impact of altitude on dairy milk composition. JDS Commun..

[57] Leiber F., Kreuzer M., Leuenberger H., Wettstein H. (2006). Contribution of diet type and pasture conditions to the influence of high altitude grazing on intake, performance and composition and renneting properties of the milk of cows. Anim. Res..

[58] Almeida M.R., De S Oliveira K., Stephani R., De Oliveira L.F.C. (2011). Fourier-transform Raman analysis of milk powder: A potential method for rapid quality screening. J. Raman Spectrosc..

[59] Khan H.H., McCarthy U., Esmonde-White K., Casey I., O’Shea N. (2023). Potential of Raman spectroscopy for in-line measurement of raw milk composition. Food Control.

[60] Zhang Z., Li S., Sha M., Liu J. (2021). Characterization of Fresh Milk Products Based on Multidimensional Raman Spectroscopy. J. Appl. Spectrosc..

[61] Yazgan N.N., Genis H.E., Bulat T., Topcu A., Durna S., Yetisemiyen A., Boyaci I.H. (2020). Discrimination of milk species using Raman spectroscopy coupled with partial least squares discriminant analysis in raw and pasteurized milk. J. Sci. Food Agric..

[62] Song S.W., Jeong Y.C., Park C.R., Kim H.M. (2023). Quantitative Fat Analysis of Milk Using a Line-Illumination Spatially Offset Raman Probe through Carton Packaging. Analyst.

[63] Zhang Z.-Y., Su J.-S., Xiong H.-M. (2025). Technology for the Quantitative Identification of Dairy Products Based on Raman Spectroscopy, Chemometrics, and Machine Learning. Molecules.

[64] AOAC International (2005). Official Methods of Analysis.

[65] Saura-Calixto F., Serrano J., Goñi I. (2006). Intake and bioaccessibility of total polyphenols in a whole diet. Food Chem..

[66] Kim D., Lee K.W., Lee H.J., Lee C.Y. (2002). Vitamin C Equivalent Antioxidant Capacity (VCEAC) of Phenolic Phytochemicals. J. Agric. Food Chem..

